# Written drug promotion from pharmaceutical companies to general practitioners: adherence to regulations over two decades

**DOI:** 10.1007/s00228-025-03951-2

**Published:** 2025-12-19

**Authors:** Gjermund Almås, Martin Stangebye, Lars Slørdal, Olav Spigset

**Affiliations:** 1 Department of Clinical and Molecular Medicine, Faculty of Medicine and Health Sciences, Norwegian University of Science and Technology, Trondheim, Norway; 2 Department of Clinical Pharmacology, St. Olav University Hospital, Trondheim, Norway

**Keywords:** Drug marketing, Drug regulation, Pharmaceutical companies, General practitioner

## Abstract

**Purpose:**

Previous studies have questioned the integrity of drug promotional material from the pharmaceutical industry to medical professionals. Herein, we present the result of repeated monitoring of adherence to legal requirements regulating marketing mailings from pharmaceutical companies to Norwegian general practitioners over three separate time periods in 2004, 2012 and 2023-24.

**Methods:**

The claims made in the mailings were compared to the sources cited and evaluated in accordance with the governmental regulations. Pertinent information about conflicts of interest was registered and considered.

**Results:**

A considerable decline in the number of mailings acquired over time was noted. The proportion of claims defined as correct and clinically relevant according to the legal definition was 48%, 56% and 78% in 2004, 2012 and 2023-24, respectively (*p* < 0.001 for trend). The most common deviations were claims with unclear or no clinical relevance, information extrapolated beyond the actual content of the referenced material, claims not covered in the cited reference, and general claims not providing any meaningful information about the drug. In some cases, the citation of sources was incomplete, or the reference unobtainable. The percentage of references with declared conflicts of interest was relatively stable at about 60% over the entire period.

**Conclusion:**

Concomitant with a decline in the number of drug mailings aimed at general practitioners, there has been an increase in compliance with legislative measures regulating this activity. Nevertheless, there are still significant instances of deviations from the legal requirements, warranting skepticism.

Marketing medicines by sending mails directly to physicians is one of several ways by which pharmaceutical companies promote their products. There is evidence to suggest that interactions between prescribers and the pharmaceutical industry are associated with increases in prescribing rates, reduced prescribing quality and increased costs [1–3]. Specifically, new drugs do not necessarily provide added therapeutic value [4] and are more expensive than older comparators. A study from 2017 of the most extensively marketed drugs in the United States concluded that the products generally were of limited therapeutic value [5]. Correspondingly, a Canadian study published the same year found that most of the expenses incurred by pharmaceutical companies for promotion were related to drugs with little or no therapeutic benefit [6]. A Danish study published in 2021 reported that most drugs being advertised in a national medical journal did not add substantial benefits over older treatments, but increased costs [7]. Nevertheless, in a survey general practitioners (GPs) in Norway reported that industry-generated information provided important decision support for the use of new medications [8].

In 1988, the World Health Organization (WHO) established *Ethical criteria for medicinal drug promotion* [9]. These criteria have been adopted and refined to provide national government regulations in many countries as well as legislation in the European Union (EU) [10]. However, in other countries, drug promotion is self-regulated by the pharmaceutical industry or is regulated by non-governmental organizations [3]. The current EU legislation is based on the *European Union (EU) council directive 92/28/EEC of March 1992 on the advertising of medicinal products for human use* [11]. In 2001 this directive was substituted by *Directive 2001/83/EC on the Community code relating to medicinal products for human use*, with the content related to drug advertising (Article 91–92) unchanged [10]. In Norway, the relevant legislation is found in Chap. 13 of the *Regulation on medicinal products for humans* [12] (Box 1). This legislation is, according to the *European Economic Area Agreement between Norway and the European Union* [13], fully compliant with EU legislation.

**Table:** Box 1 Extracts from the Norwegian *Regulation on medicinal products for humans*, Chapter 13, *Advertising of medicinal products* [12]. (Translated from Norwegian)

**§ 13-3** **:**
[...] Promotion of a medicinal product shall comply with the information in the Summary of product characteristics approved by the Norwegian Medical Products Agency. It shall
a. promote the rational use of the medicinal product by presenting it objectively without exaggerating its properties, and
b. not be misleading or deceptive.
**§ 13-8:**
[...] All information [...] shall be accurate, up-to-date, verifiable, accessible to the recipient, and sufficiently detailed to enable the recipient to form his or her own opinion of the therapeutic value of the medicinal product.
Quotations, tables, and other illustrations taken from medical journals or scientific works shall [...] be reproduced faithfully with precise source attribution.
Medical journals shall undergo professional quality assurance (peer review). Scientific works shall undergo professional quality assurance (peer review) and be published.

Numerous studies have assessed the quality of drug advertising in medical journals. Outcome measures in these studies include validity and clinical relevance of claims, risk reduction presentations, figure quality, and availability and quality of the cited references [14]. A systematic review from 2009 on advertisements in medical journals from 26 countries concluded that the quality of journal advertising material was generally low and represented a global challenge [14]. In seven of the studies included in this review, compliance with national codes, guidelines or regulations were investigated. Notably, all these studies were published between 1989 and 2003.

In contrast to the situation for drug advertising in medical journals, very few studies have investigated the quality of information in direct-to-physician mailings. Moreover, there is generally a lack of comprehensive studies of promotional materials over time and a paucity of recent studies. The aim of the present study was to investigate whether direct marketing material from pharmaceutical companies to GPs in Norway complied with the national drug marketing legislation over the last 20-year-period.

## Methods

Direct marketing material sent by mail from pharmaceutical companies to a selection of GPs in Norway were collected over three time periods, 1 September – 30 November 2004, 1 June – 31 August 2012 and 1 October 2023 – 31 March 2024. Findings from the two first periods have been published separately in the Journal of the Norwegian Medical Association [15, 16]. The number of GPs and GP offices involved in the collection are presented in Table 1. Due to the impression that the number of mailings received was reduced over time, the number of GPs involved and the collection periods were gradually increased over time to collect fairly equivalent numbers of mailings in total.

**Table 1:** Data collection and number of mailings, claims, and references in the three study periods 2004 [15], 2012 [16] and 2023-24

	2004	2012	2023-24
Data collection			
Number of GPs collecting data	3	9	15
Number of GP offices participating	2	2	3
Length of collection period (months)	3	3	6
Number of mailings received	355	270	275
Number of drugs advertised and selected for the study	50	46	23
Claims			
Total number of claims	261	156	78
Literature references			
Total number of references cited	191	173	112
Reference to unpublished material / data on file	10 (6%)	4 (2%)	0 (0%)
Unclear/incomplete reference citations	3 (2%)	5 (3%)	4 (4%)
References identified and obtained	178 (92%)	164 (95%)	108 (96%)
Obtained references			
Ordinary journal articles	121 (68%)	115 (70%)	58 (52%)
Articles in journal supplements	25 (14%)	6 (4%)	0 (0%)
Summary of product characteristics	15 (8%)	30 (18%)	38 (34%)
Textbooks, websites, guidelines, congress material	17 (10%)	13 (8%)	16 (14%)

In cases where multiple mailings were received for a single drug, we chose to evaluate the mailing with the highest number of references. If a mailing promoted multiple medications, these medications were evaluated individually.

After collection and selection, the references cited in the included texts were acquired. In cases where the references consisted of more than just the Summary of Product Characteristics (SmPC) and other generally accessible sources, the pharmaceutical companies were contacted via e-mail to provide the references. If a company did not respond within a week, a follow-up e-mail was dispatched. If there was still no response, searches were conducted via PubMed, and, if still unsuccessful, we called upon the assistance from the staff at the Medical Library at The Norwegian University of Science and Technology (NTNU). The claims were checked for consistence with the content in the cited articles, and the articles were also scrutinized for information on the authors’ conflicts of interest. Claims without any cited references were evaluated according to the text in the SmPC for the drug.

The material was categorized according to the requirements in the Norwegian *Regulation on medicinal products for humans*, Chap. 13 [12]. Although the regulation related to drug promotion and advertising has been updated since the first collection period in 2004, the content related to the quality of written drug information is for all practical purposes identical; the updates have solely been of technical nature. The categories chosen to classify the material are presented in Table 2. “Inaccurate” claims exaggerated the properties of the drug in question or left out obviously relevant information. The “meaningless” category included, for example, claims such as “a real advantage”, “a major step forward” and similar phrases. Additional examples of how claims were categorized are given in the footnote to Table 2. These included the extent to which the claims in the texts matched the content in the references cited. The initial evaluation and categorization were done by the first two authors of the two previously published articles [15, 16] as well as of the present one. In cases of disagreement, all four authors discussed the issue until consensus was achieved. We did not make any formal attempts to ensure internal validity, nor did we perform any evaluations of the degree of inter-rater agreement.

**Table 2:** Claim categories and number of incorrect/non-relevant claims classified according to the Norwegian regulation on medicinal products for humans (Box 1) in the three study periods 2004 [15], 2012 [16] and 2023-24

Category	Description	2004 (*n* = 261)	2012 (*n* = 156)	2023-24 (*n* = 78)
Correct	The information is accurately reproduced from the source and is clinically relevant	126 (48%)	87 (56%)	61 (78%)
Correct, but without clinical relevance	The information is correctly reproduced, but the claim is of dubious or no clinical relevance^a^	27 (10%)	22 (14%)	4 (5%)
Inaccurate	The information is extrapolated beyond what the reference suggests, portraying the medication in a more favorable light than warranted, or there are evident omissions of relevant information	35 (13%)	12 (8%)	3 (4%)
False	Undisputable misinformation compared to data cited in the reference or in the medication’s Summary of product characteristics	15 (6%)	7 (4%)	0 (0%)
Not covered in the cited reference	The claim is not mentioned in any of the provided references	15 (6%)	4 (3%)	3 (4%)
Meaningless	The claim does not give any substantial information about the medication, or is of such general nature that it cannot be proven or disproven	13 (5%)	15 (10%)	3 (4%)
Unavailable reference	At least one of the references cited to substantiate the claim could not be identified/retrieved	9 (4%)	1 (1%)	4 (5%)
Lack of reference	The claim lacks a reference and is not supported by the medication’s Summary of product characteristics	21 (8%)	8 (5%)	0 (0%)

^a^ Causes for a claim to be classified into this group included: (a) the underlying study did not investigate “hard” endpoints (e.g., morbidity, hospital admissions, mortality) but only surrogate endpoints (e.g., reduction in blood glucose, HbA1c, or blood pressure), (b) the underlying study used a different dosage than recommended according to the Summary of Product Characteristics, (c) statistically significant differences were found in the underlying study, but the absolute differences were so small that they are unlikely to have any clinical relevance, (d) pharmacokinetic arguments lacking documented clinical significance were used (e.g., related to elimination half-life or degree of absorption), (e) pharmacodynamic arguments lacking documented clinical significance were used (e.g., related to mechanism of action or receptor selectivity)

Data are presented with numbers and percentages as appropriate. Trends over time were evaluated by the chi-square linear-by-linear test for trend. Two-sided p-values < 0.05 were considered to represent statistical significance. Statistical analyses were performed with IBM SPSS version 30 (IBM, Armonk, NY, USA).

## Results

The number of mailings collected was 355 in 2004, 270 in 2012 and 275 in 2023-24. The numbers of claims investigated were 261, 156 and 78, with a total of 191, 173 and 112 references cited, respectively. There was a large variation in the number of mailings received for each drug, ranging from 1 to 53. Data related to the collection, including the numbers of mailings, claims and references included, are presented in Table 1. In general, the drugs included in the analyses were representatives of major drug groups used in prevalent conditions, including hypertension, hyperlipidemia, type 2 diabetes, asthma/chronic obstructive pulmonary disease, allergic rhinitis, urinary incontinence and osteoporosis. Some examples of heavily marketed specific substances that were included in this study in each of the three study periods are shown in Table 3.

**Table 3:** Examples of substances heavily marketed and included in the study (in alphabetical order) in the three study periods 2004, 2012 and 2023-24

2004	2012	2023-24
Alendronate Amlodipine Atorvastatin Candesartan Clarithromycin Fluticasone/salmeterol Formoterol Losartan Tamsulosin Tolterodine	Beclomethasone Celecoxib Desloratadine Ezetimibe Linagliptin Methenamine Methylphenidate Olopatadine Pregabalin Tadalafil	Adapalene/benzoyl peroxide Dapagliflozin Empagliflozin Melatonin Methenamine Mometasone/olopatadine Pneumococcal vaccine Rimegepant Semaglutide Tiotropium/olodaterol

The proportions of cited claims defined as correct and clinically relevant were 48% in 2004, 56% in 2012 and 78% in 2023-24 (*p* < 0.001). An overview of the number of incorrect/non-relevant claims grouped according to categories is presented in Table 2. The reduction in the number of claims over the years seems to be roughly equally distributed across the subgroups (Table 2), although these changes were not statistically tested due to the low numbers involved.

The number of articles classified according to conflicts of interest is shown in Table 4. The percentage of articles with a declared conflict of interest was relatively stable; 61% in 2004, 75% in 2012 and 59% in 2023-24. In contrast, the percentages of articles not specifying whether there were any conflicts of interest decreased from 32% in 2004 to 15% in 2012 and 16% in 2023-24, whereas the percentage of articles explicitly stating the absence of conflicts of interest gradually increased from 7% in 2004 via 10% in 2012 to 25% in 2023-24 (*p* < 0.001).

**Table 4:** Overview of conflicts of interest in the articles obtained in the three study periods 2004 [15], 2012 [16] and 2023-24^a^

	2004 (*n* = 146)	2012 (*n* = 121)	2023-24 (*n* = 51)^b^
Articles with a declared conflict of interest	89 (61%)	91 (75%)	30 (59%)
- Articles with a financial conflict of interest	56 (38%)	90 (74%)	30 (59%)
- Articles with other conflict of interest	33 (23%)	1 (1%)	0 (0%)
Articles explicitly stating absence of conflicts of interest	10 (7%)	12 (10%)	13 (25%)
Not specified	47 (32%)	18 (15%)	8 (16%)

^a^ The total numbers represent the sum of ordinary journal articles and articles published in journal supplements, as presented in Table 1

^b^ The number is lower than in Table 1 because seven duplicate articles have been omitted

## Discussion

The principal finding in the present study is that in promotional mailings sent by the pharmaceutical industry to GPs in Norway, the proportion of claims defined as correct and clinically relevant according to Norwegian legislation increased from 48% in 2004 via 56% in 2012 to 78% in 2023-24. This represents a clearly positive trend, although there is still a fairly high number of claims that do not meet regulatory requirements. Furthermore, an important caveat is that the categorization of “correct” and “clinically relevant” is based solely upon what is stated in the cited source, irrespective of the data quality of that source and findings in additional non-cited sources. Thus, a categorization of a claim as “correct” and “clinically relevant” does not necessarily mean “objectively true”. For instance, a mailing received in 2023-24 for a product containing doxylamine and pyridoxine cited an apparently positive study from 2010 [17]. However, in 2018 data from this study were reanalyzed, showing that the method of handling missing data significantly impacted whether the results could be considered clinically significant or not [18]. Thus, the actual proportion of claims presenting a “true picture” of the therapeutic value of a drug may in fact be lower than the results from our study suggest. In general, a low adherence to basic regulations related to quotation practice would, in our view, relegate the written information investigated to a status of being of little, if any, value as information sources.

Another factor to take into consideration is that the proportion of articles with a positively declared conflict of interest was generally high throughout our study period, at about 60% or more. Analyses have demonstrated that economic conflicts of interest introduce bias in favor of the test drug with an odds ratio of up to 2–3 irrespective of medical specialty or type of drug [19]. Thus, articles still contain results that must be critically assessed in light of the reported conflicts of interest, regardless of whether the claims can be categorized as “correct” and “clinically relevant”. The proportion of articles explicitly stating that no conflicts of interest exist was 25% in 2023-24, representing an increase from 7% in 2004 and 10% in 2012. Correspondingly, the proportion of articles without any statement of whether a conflict of interest exists or not decreased during the period. The reason may be that medical journals now often require that a declaration form with explicit and specific information related to any potential conflicts of interest should be included. Nevertheless, there are still high proportions of incorrect conflict of interest disclosures, including undisclosed financial connections, in medical journals [20, 21]. We made no attempts to check the veracity of the declared conflicts of interest in the referenced literature. It should also be noted that citations to narrative or systematic review articles might present misleading information, as the relevant conflict of interest data in such cases would be found in the underlying original studies. The decrease from 14% to 0% regarding references to journal supplements is a desired change, as such publications are more rarely subjected to peer review.

In 2004 and 2012, the proportion of unequivocally false claims was 6% and 4%, respectively. For example, in the 2004 material we found a claim that a topical drug containing azelaic acid was “without any dermal irritation”. However, in the 2004 SmPC application site burning, pain, and pruritus were listed as very common adverse reactions. Similarly, in the 2012 material we identified a claim that a topical drug containing capsaicin was “completely without systemic adverse effects”, although nausea, muscle spasms and hypertension, among others, were listed in the SmPC. In contrast, there were no false claims identified in the 2023-24 material, which is notable.

The number of claims that were correct and clinically relevant was higher in our 2023-24 material than in studies from other countries during the last 10-year period [22–25]. In contrast, the proportions of correct and clinically relevant statements in our analyses of 2004 and 2012 mailings were more in line with what was seen internationally during those time periods [14, 26–28]. However, the concomitant decline in the actual number of mailings over time (Table 1) may suggest that focus on fewer drugs facilitates industry’s legislative compliance.

The quality of written drug information may be related to the national regulatory control systems, including national legislation, enforcement by regulatory authorities and initiatives from the national pharmaceutical organizations [24]. In Norway, drug marketing legislation has not been altered throughout the 20-year period, but the resources allocated to enforcement by the regulatory authorities have increased. It is now compulsory for the marketing authorization holder of a drug to send all promotional material to the Directorate for Medical Products before dispersal. The pharmaceutical industry itself has also taken measures. The Association of the Pharmaceutical Industry in Norway has developed a comprehensive guideline with industry rules related to marketing [29] and also arranges educational courses for key personnel. Finally, The Council for Pharmaceutical Information has been jointly established by The Association of the Pharmaceutical Industry in Norway and The Norwegian Medical Association as an independent self-regulatory body for pharmaceutical companies [30].

This study has some limitations that warrant attention. Firstly, all assessments made in this study have been subjective. We did not make any formal attempts to ensure internal validity, nor did we perform any evaluations of the degree of inter-rater agreement. However, the fact that two of the four investigators in the three surveys that together constitute the present work have remained unchanged may represent a kind of continuity which to some degree may remedy this. Secondly, it should be noted that relatively small samples of mailings and claims have been analyzed and that the degree of generalizability of our findings therefore could be limited. Thirdly, we have only examined some selected quality aspects, although our evaluation has the advantage of being based on the legislation in the field. Aspects that we have not considered include the use of illustrations and other persuasion strategies in the marketing material [31]. Fourthly, the conflicts of interest part of our study has inherent weaknesses, both due to the expected extent of underreporting and because when review articles were cited, conflicts of interest information in that article and not in the relevant underlying original article was registered. Finally, the study only investigated one platform of written information, which in the present day and age seems to be in the process of being replaced with other marketing modalities.

In conclusion, there was a considerable decline in the number of mailings acquired over time. In parallel, the proportion of claims defined as correct and clinically relevant according to the legislative definition increased from 48% in 2004 via 56% in 2012 to 78% in 2023-24. The most common deviations were claims with unclear or no clinical relevance, information extrapolated beyond what the reference suggested, claims not covered in the cited reference, and general claims not providing any meaningful information about the drug. Although there has been a clear increase in compliance with legislative measures during the period studied, there are still significant instances of deviations from the legal requirements.

## Data Availability

The dataset generated and analyzed during the current study is available from the corresponding author upon reasonable request.
